# Effects of Music Therapy on Pain, Anxiety, and Quality of Life During Lower Third Molar Extraction: A Randomized Double‐Blind Clinical Trial

**DOI:** 10.1155/ijod/2182937

**Published:** 2026-10-13

**Authors:** Antônio Asriel dos Santos Almeida, Bárbara Albuquerque Azevedo, Giselly dos Santos Gomes, Mara Bianca Campos de Araújo, Maria Cecília Dantas Gadelha, Bruna Larissa dos Santos Pinto, Isadora Idelfonso Monteiro Rodrigues, Rebeca Freitas Lima, Ana Rebeca Fernandes da Silveira, Marina Gurgel Tavares Guerra, Milena Alves Coutinho, Julianne Coelho da Silva, Rosária Emanuelly Oliveiras Bonfim, Paulo Goberlânio de Barros Silva, Edson Luiz Cetira Filho

**Affiliations:** ^1^ Postgraduate Program in Dentistry UNICHRISTUS Fortaleza Ceará Brazil unichristus.edu.br; ^2^ Academic of Dentistry Christus University (UNICHRISTUS) Fortaleza Ceará Brazil unichristus.edu.br; ^3^ Department of Dentistry University of Fortaleza (UNIFOR) Fortaleza Brazil unifor.br; ^4^ Department of Dentistry Christus University (Unichristus) Fortaleza Ceará Brazil unichristus.edu.br

**Keywords:** anxiety, molar, music therapy, pain, surgery, third

## Abstract

Third molar extractions are commonly associated with anxiety, pain, and a negative impact on oral health‐related quality of life, potentially compromising patient recovery and overall well‐being. Nonpharmacological approaches, such as music therapy, have been investigated as adjunctive strategies to improve perioperative outcomes. This randomized, blinded, controlled clinical trial evaluated the effects of music therapy in 42 patients undergoing third molar extraction, who were allocated into two groups: with and without music therapy. The primary outcomes were pain, assessed using the visual analog scale, and anxiety, measured using the *Dental Anxiety Scale*, while oral health‐related quality of life, evaluated using the *Oral Health Impact Profile* (OHIP‐14), was considered a secondary outcome. The control group showed higher systolic blood pressure and respiratory rate. Pain levels increased intraoperatively and decreased postoperatively in both groups, with lower immediate postoperative pain scores observed in the music therapy group. No significant differences were found regarding anxiety levels or quality‐of‐life outcomes. These findings suggest that music therapy may contribute to the reduction of physiological parameters, such as blood pressure and respiratory rate, as well as participants pain perception, supporting its use as a complementary nonpharmacological strategy in third molar extraction procedures.

## 1. Introduction

Numerous patients need to undergo third molar extractions. These elements are chronologically the last to erupt into the oral cavity and, therefore, are often retained by soft, hard tissues, or both, which can cause a series of problems for the patients oral health [1].

It is undeniable that surgical procedures, although they have evolved considerably, can be traumatic, especially when patients experience psychosomatic problems, resulting in an unpleasant emotional experience [2]. In response to these undesirable stimuli, the human physiological system responds negatively, increasing blood pressure, respiratory rate, and heart rate [3].

Patients who present symptoms of anxiety tend to cooperate less. Therefore, dentists usually prescribe anxiolytic medications to assist in the appropriate management of individuals [4]. These medications enhance neurotransmitters that inhibit the central nervous system. Therefore, studies have indicated music therapy as an adjunctive approach for the management of psychogenic factors through a nonpharmacological intervention [5].

Keilani et al. [6] state that “the combination of pharmacological and non‐pharmacological methods in managing anxiety and pain could probably have a synergistic effect to improve pain relief for the patient.” Some studies suggest using music therapy as a nonpharmacological method, effectively aiding psychological control when exposed to less comfortable environments. Music, when listened to, activates areas of the brain and can release dopamine, a neurotransmitter capable of generating feelings of pleasure and motivation. “The effect of music on the autonomic nervous system has been studied for a long time; however, there is no consensus on the effect of music on the autonomic nervous system” [7].

Thus, this study aimed to identify the effects of using music therapy in third‐molar removal surgeries to reduce patients anxiety and pain during dental treatment, in addition to the impact on the quality of life of the respective patients.

## 2. Materials and Methods

### 2.1. Ethical Considerations

This work followed the Health Research Standards of the Christus University Center and the Brazilian National Health Council (Resolution number 466 of 2012). Following the Helsinki Declaration, the research protocol was submitted to the Ethics Committee on Research in Human Beings of the Centro Universitário Christus/IPADE number 4,928,668 for approval. The volunteers of the present study received an informed consent form, in which the objectives, methodology, and related risks and benefits were explained, and their confidentiality was respected.

### 2.2. Study Design

A randomized, prospective, blind, controlled, and analytical clinical trial was conducted. It was registered in the Brazilian Registry of Clinical Trials (ReBEC), under number: RBR‐105fptdt. In addition, this clinical trial followed the items described in the latest version of the *“Consolidated Standards of Reporting Trials, ”*CONSORT (Figure 1).

**Figure 1:**
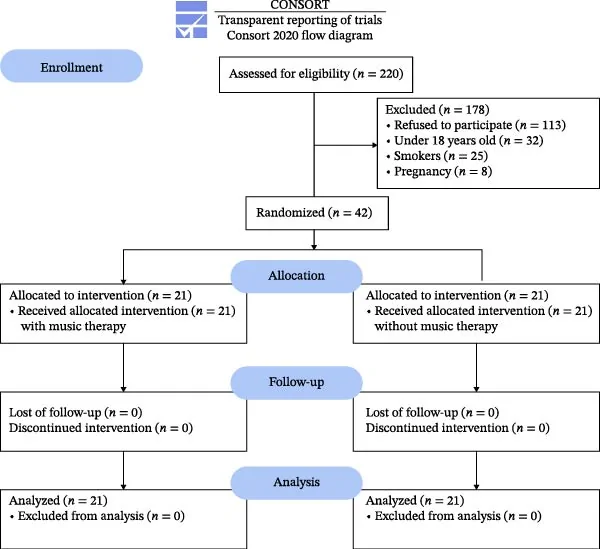
Consolidated standards of reporting trials: flow diagram.

### 2.3. Research Participants

Individuals recruited from the spontaneous demand of the Oral Surgery Service of the Dentistry course of the Christus University Center, requiring extraction of mandibular third molars, were invited to participate in this study. The volunteers were initially evaluated regarding the eligibility criteria that guided the selection of participants for this clinical trial based on those adopted by Albuquerque et al. [8]. An appropriate clinical chart was completed during the initial and postoperative consultations [9].

Healthy subjects (ASA I) of both genders, aged between 18 and 35 years, requiring removal of mandibular third molars, were invited to participate in this study. In addition, the following inclusion criteria were adopted: (1) retained mandibular third molars and (2) third molars presenting similar patterns of root formation, position, and degree of impaction on panoramic radiographs when comparing the teeth of each volunteer, aiming to maintain a similar degree of operative trauma. Only third molars classified as Pell and Gregory Class II‐B and Nolla stage 10 were included.

Volunteers who met at least one of the following criteria were excluded: (1) smokers; (2) pregnant or lactating women; (3) carriers of orthodontic bands on mandibular second molars; (4) signs of any preoperative inflammatory or infectious condition; (5) systemic diseases; (6) neurological disorders; and (7) patients without hearing or with auricular disorders referring to the interpretation of sounds (sound frequencies), which may influence the outcomes of the present study Albuquerque et al. [8].

Removal criteria that were considered during the development of the study were (1) patients who did not follow the postoperative recommendations; (2) surgical procedures that exceeded 1 h; or also, (3) patients who did not return for the postoperative evaluation appointments.

### 2.4. Research Interventions

In a presurgical procedure consultation, data were collected regarding gender, age, general health status, in addition to requesting hematological tests and imaging tests.The following data were collected: degree of tooth development (Nolla stages), level of third molar impaction, and third molar position according to Pell and Gregory and Winter’s classification. All patients were submitted to a standardized surgical technique supported by scientific evidence, performed in an outpatient setting under local anesthesia, following strict biosafety control. A single maxillofacial surgeon with 6 years of experience in the respective specialty performed all surgical procedures.

The removal of the mandibular third molars was performed under local anesthesia with mepivacaine 2% and epinephrine 1:100,000 (Mepivalem AD; Dentsply, USA), using a total of two cartridges containing 1.8 mL of the anesthetic solution in each. A full‐thickness triangular flap was performed, and it may be necessary to perform peripheral ostectomy or odontosection. Ultimately, the surgical wound was closed using a 4‐0 silk thread suture.

Individuals deemed eligible to participate in the present study were randomly allocated, using a list of random numbers generated in Microsoft Excel with the “RANDBETWEEN” function [8], into one of two groups according to the use or nonuse of music therapy during the trans‐surgical period. Both groups wore headphones throughout the procedure; however, in the control group, the headphones remained inactive. During the initial consultation, each volunteer selected one of the following musical genres: Brazilian Popular Music (BPM), romantic rock, punk rock, instrumental music, Brazilian pagode, country, Brazilian forró, classical music, or another genre. The selected musical style was played throughout the entire surgical procedure through Beats Studio Pro headphones (Beats Electronics, LLC, Cupertino, CA, USA).

In the postoperative period, all volunteers instructed to use rescue medication if necessary. Dipyrone 500 mg (at 6‐h intervals) was prescribed because it is recommended as rescue medication in a similar methodology to this one by Barbalho et al. [10]. Postoperative medication was prescribed according to the following instructions: in case of pain, patients were instructed to initiate treatment with dipyrone 500 mg every 6 h and discontinue its use once the pain had subsided. Postoperative instructions were also read and explained carefully. Patients were informed to contact the surgeon initially by phone in case of persistent bleeding or if deemed necessary. In addition, patients were also asked to report any physical symptoms experienced during the postoperative periods of the study, such as nausea, vomiting, dizziness, headache, insomnia, and signs of infection. In cases where an infectious process was found to be present, amoxicillin 500 mg (at 8‐h intervals for 7 days) was prescribed.

### 2.5. Research Outcomes

The primary outcomes were changes in anxiety levels and parameters related to postoperative pain. The secondary outcomes were the assessment of vital signs during the preoperative, intraoperative, and postoperative periods, as well as the impact on quality of life during the first postoperative week.

#### 2.5.1. Postoperative Pain Assessment

The outcome measurement related to postoperative pain was performed by assessing pain intensity and the need for rescue medication. The volunteers were duly instructed to use a 10‐cm visual analog scale (VAS), which consists of a scale with values ranging from 0 (no pain/discomfort) to 10 (maximum pain/discomfort) [10–12].

Before the surgical procedure, each patient received explanations on how to indicate pain intensity through the VAS. After the surgical procedure, each patient received a form so that he/she could write down the postoperative pain scores, and the referred document was returned to the researcher on the day of suture removal (seventh postoperative day). In this sense, the volunteers of this study were asked to inform the scores of pain intensity in the following periods of postoperative evaluation: 0 (end of the surgical procedure), 12 and 24 h after surgery; this information was collected via the “*WhatsApp*” application in the periods in which there would be no face‐to‐face consultation. Additionally, the form that each patient received had a space so that he/she could write down the time needed to take the first rescue medication (if necessary) as performed by Costa et al. [11] as well as the daily consumption of rescue medications that patients needed to take during the postoperative period.

#### 2.5.2. Anxiety Scale

The dental anxiety scale (DAS) questionnaire was applied to observe patients anxiety levels. The questionnaire was applied preoperatively and postoperatively and was composed of questions that assessed the prevalence and behavior of the individual in the face of surgery for mandibular third molar exodontia, such as whether they had already had an exodontia and how often they avoided and missed appointments because of fear.

#### 2.5.3. Vital Signs

Vital signs were assessed at three time points: preoperatively, intraoperatively (immediately after tooth sectioning), and postoperatively (immediate postoperative period). Systolic and diastolic blood pressure and heart rate were measured using a digital upper‐arm monitor (model HEM‐7156T, Omron Healthcare, Kyoto, Japan), employing the oscillometric method and Intellisense technology, in accordance with current calibration guidelines. Body temperature was assessed using an Omron digital thermometer (model MC‐246). Peripheral oxygen saturation (SpO_2_) was assessed using noninvasive digital pulse oximetry with a portable fingertip pulse oximeter (Rossmax SB100/MD), which was previously inspected to ensure sensor integrity and adequate battery charge. The index (or middle) finger of the dominant hand was cleaned and inserted into the oximeter. To ensure measurement reliability, nail polish or artificial nails were removed from participants before assessment.

#### 2.5.4. Quality of Life Assessment

To evaluate the quality of life, each patient received a questionnaire to be answered, named “Oral Health Impact Profile” (OHIP‐14), which was focused on adverse effects [13]. Each patient was required to answer the questionnaires at the initial visit, 24 h after the surgical procedure, and at the end of 7 days postoperatively.

The OHIP‐14 questionnaire consists of 14 questions distributed among seven domains: functional limitation, physical pain, psychological discomfort, physical impairment, psychological impairment, social disability, and social disadvantage. Two questions were applied to each of these domains. In addition, delayed clinical recovery was considered if the patient returned and was found to require the institution of one or more of the following four procedures: prescription of an antibiotic or analgesic, reopening of the wounded surgery, debridement of the surgical wound, or placement of sterile gauze at the surgical site [14]. Prevalence, extent, and severity scores were calculated preoperatively and postoperatively according to the methodology described by Shugars et al. [13]. The percentage of volunteers who reported one or more items “fairly” or “very often” represented a measure of prevalence. An item reported as “fairly often” or “very often” was considered clinically important and detrimental to the quality of life. In addition, each subject’s extent score was computed as the number of items reported “fairly often” or “very often,” while severity was computed as the sum of OHIP‐14 scores, coded as follows: never? (0 points), rarely? (1 point), occasionally? (2 points), fairly often? (3 points), and often? (4 points).

The questionnaires were administered by a collaborator who did not know the group or subgroup to which the patient belonged and who would not participate in the surgical procedures or even in the measurement of the other outcomes, thus allowing the blinding of the study.

### 2.6. Sample Size

Based on the study by Keilani et al. [6], who noted that in patients who underwent music intervention for anxiety and pain reduction in outpatient maxillofacial and ENT surgery, there was a significant reduction in anxiety levels (3.1 ± 2.3 to 1.21 ± 0.85). After surgery, it was estimated to be necessary to evaluate a sample of 18 patients per study group using a power of 90% and a confidence level of 95%. Considering the possibility of sample loss in the postoperative evaluation period, 10% was added to the sample, totaling 20 patients per group defined in this study.

### 2.7. Blinding

To ensure the blinding of the surgeon regarding the intervention, the surgeon was unaware of the group to which each volunteer had been allocated and, therefore, did not know which participants would undergo music therapy. A second researcher, who was not involved in the surgical procedures, was responsible for organizing the participant allocation flow according to the previously established randomization sequence and directing participants to their respective groups. Thus, the surgeon did not have access to the randomization sequence or group identification during the procedures. Additionally, to ensure the blinding of the statistician, the researcher responsible for the statistical analysis was unaware of the group to which each volunteer belonged [15]. The collaborator entered the collected data into an electronic spreadsheet, with the groups in the present study coded as “A” and “B” (music and no music, respectively) to ensure statistician blinding. Statistical analysis was initially performed using the coded groups, and the codes were revealed only after the results had been obtained, thereby ensuring double blinding (Surgeon and statistician).

### 2.8. Statistical Methods

Data were tabulated in Microsoft Excel version 2020 and exported to IBM software SPSS Statistics for Windows, version 20 (IBM Corp., Armonk, NY, USA), in which analyses were performed adopting a 95% confidence level.

Between‐group and within‐group analyses were performed. All categorical data were compared between groups using Fisher’s exact test or Pearson’s chi‐square test. Quantitative data were subjected to the Kolmogorov–Smirnov normality test and compared between groups using the Mann–Whitney test and within groups (temporal analysis) using the Friedman test followed by Dunn’s post hoc test. Additionally, Kaplan–Meier curves were used to analyze the time to first rescue medication, with comparisons performed using the Mantel–Cox log‐rank test. For the assessment of the time × group interaction, a two‐way repeated‐measures ANOVA was used for parametric data, while a generalized linear model (GLM) was used for nonparametric data.

## 3. Results

Odontectomy and osteotomy were performed in all cases, following a standardized surgical protocol. Participant inclusion was based on the Pell and Gregory Class II‐B classification, determined after panoramic radiographic assessment, in order to ensure greater uniformity among cases and reduce potential variables related to surgical complexity and technique.

Most of the sample were women in both groups (*p* = 0.352), with a mean age of 23.64 ± 3.58 and 23.55 ± 3.24 (*p* = 0.879), respectively, and mean weight of 76.59 ± 17.56 and 68.20 ± 12.03 (*p* = 0.154). There was no significant difference in clinical characteristics between the two study groups (Table 1).

**Table 1:** Personal data and clinical characteristics of the patients.

Patient data	Group		*p*‐Value
	Control	Music therapy	
Sex			
Female	10 (47.6%)	13 (61.9%)	0.352
Male	11 (52.4%)	8 (38.1%)	
Age	23.64 ± 3.58	23.55 ± 3.24	0.879
Weight	76.59 ± 17.56	68.20 ± 12.03	0.154
Current medical treatment	0 (0.0%)	1 (4.8%)	0.311
Current medication	2 (9.5%)	2 (9.5%)	1.000
Medication last 15 d	3 (14.3%)	1 (4.8%)	0.293
Allergy	4 (19.0%)	3 (14.3%)	0.679
Surgery	6 (28.6%)	9 (42.9%)	0.334
Healing problem	1 (4.8%)	1 (4.8%)	1.000
Hemorrhage	0 (0.0%)	0 (0.0%)	1.000
Family health problem	12 (57.1%)	11 (52.4%)	0.757
Pregnancy	0 (0.0%)	0 (0.0%)	1.000
Breastfeeding	0 (0.0%)	1 (4.8%)	0.311
Contraceptive	4 (19.0%)	2 (9.5%)	0.378
Disease	1 (4.8%)	3 (14.3%)	0.293
Smoker	0 (0.0%)	0 (0.0%)	1.000
Alcoholism	5 (23.8%)	1 (4.8%)	0.078
Narcotics	0 (0.0%)	0 (0.0%)	1.000
Analgesic	0 (0.0%)	0 (0.0%)	1.000

*Note:* *p* < 0.05, Fisher’s exact test or Pearson’s chi‐square test (*n*, %).

All volunteers presented with no baseline pain prior to surgery (VAS = 0); Group with Music Therapy: 0.10 ± 0.33 (*p* = 1.000) and Group without Music Therapy: 0.10 ± 0.47 (*p* = 1.000).

The mean surgical time did not differ significantly between the groups without and with music therapy (10.54 ± 0.24 vs. 10.65 ± 0.53, respectively) (*p* = 0.508).

The prospective memory profile QAMP showed no significant difference between the groups. Both the control group and the music therapy group showed a high frequency of QAMP1 (*p* = 0.311) and QAMP2 (*p*  > 0.05). There was no difference in QAPM3 (*p* = 0.204), QAPM4 (*p* = 1.000), QAPM5 (*p* = 0.293), and QAPM6 (*p*  > 0.05) (Table 2).

**Table 2:** Prospective memory profile.

Profile	Group		*p*‐Value
	Control	Music therapy	
Do you like to listen to music?	20 (95.2%)	21 (100.0%)	0.311
What kind of music do you like to listen to?	2.14 ± 1.52	2.85 ± 1.53	0.088
Brazilian Popular Music (BPM)	10 (47.6%)	14 (66.7%)	0.212
Romantic rock	3 (14.3%)	4 (19.0%)	0.679
Punk rock	1 (4.8%)	3 (14.3%)	0.293
Instrumental	0 (0.0%)	2 (9.5%)	0.147
“Brazilian Pagode”	7 (33.3%)	6 (28.6%)	0.739
Country	7 (33.3%)	6 (28.6%)	0.739
“Brazilian Forró”	5 (23.8%)	9 (42.9%)	0.190
Classic	2 (9.5%)	6 (28.6%)	0.116
Other	11 (52.4%)	8 (38.1%)	0.352
Do you have a favorite singer?	15 (71.4%)	11 (52.4%)	0.204
Do you think listening to music can relax you?	21 (100.0%)	21 (100.0%)	1.000
Would you like to listen to music when you are undergoing dental treatment?	18 (85.7%)	20 (95.2%)	0.293
Is there any kind of music you do not like?	2.64 ± 2.06	2.30 ± 1.38	0.906
Brazilian Popular Music (BPM)	3 (14.3%)	2 (9.5%)	0.634
Romantic rock	7 (33.3%)	6 (28.6%)	0.739
Punk rock	13 (61.9%)	10 (47.6%)	0.352
Instrumental	10 (47.6%)	4 (19.0%)	0.051
“Brazilian Pagode”	5 (23.8%)	8 (38.1%)	0.317
Country	5 (23.8%)	8 (38.1%)	0.317
“Brazilian Forró”	2 (9.5%)	4 (19.0%)	0.378
Classic	7 (33.3%)	5 (23.8%)	0.495
Other	4 (19.0%)	1 (4.8%)	0.153

*Note:* *p* < 0.05, Fisher’s exact test or Pearson’s chi‐square test (*n*, %).

Regarding the signs of transoperative stress, the control group (*p* = 0.569) and the music therapy group (*p* = 0.247) showed no significant variation in systolic or diastolic pressure (*p* = 0.719 and *p* = 0.482, respectively). However, the mean systolic pressure of the control patients was significantly higher than the music therapy patients (*p* = 0.021). The differential pressure showed no significant variation from preoperative to postoperative, but in the music therapy group, there was a significant increase transoperatively and postoperatively (*p* = 0.030). There was no difference between the groups (Table 3).

**Table 3:** Vital signs of the patients.

Patient profile	Group		*p*‐Value ^∗^	Group	Time	Group vs. time
	Control	Music therapy				
Systolic pressure						
Presurgical	124.36 ± 15.39^Aa^	119.65 ± 12.24^Aa^	0.338	***0.018***	0.347	0.222
Trans‐surgical	127.23 ± 14.99^Aa^	123.80 ± 14.15^Aa^	0.545	—	—	—
Postsurgery	129.14 ± 16.29^Aa^	118.70 ± 13.59^Ba^	***0.021***	—	—	—
*p*‐Value ^∗∗^	0.569	0.247	—	—	—	—
Diastolic pressure						
Presurgical	74.64 ± 20.50^Aa^	73.90 ± 17.49^Aa^	0.830	0.056	0.453	0.771
Trans‐surgical	79.59 ± 7.19^Aa^	75.00 ± 12.11^Aa^	0.267	—	—	—
Postsurgery	69.41 ± 28.18^Aa^	70.25 ± 26.63^Aa^	0.940	—	—	—
*p*‐Value ^∗∗^	0.719	0.482	—	—	—	—
Differential pressure						
Presurgical	49.73 ± 22.65^Aa^	45.75 ± 21.54^Aa^	0.465	0.509	0.408	0.401
Trans‐surgical	47.64 ± 16.67^Aa^	48.80 ± 13.11^Ab^	0.554	—	—	—
Postsurgery	59.73 ± 35.21^Aa^	48.45 ± 28.65^Ab^	0.194	—	—	—
*p*‐Value ^∗∗^	0.566	***0.030***	—	—	—	—
Heart rate						
Presurgical	78.36 ± 10.69^Aa^	79.75 ± 13.45^Aa^	0.970	0.192	0.252	***0.047***
Trans‐surgical	88.05 ± 19.55^Ab^	82.65 ± 10.47^Aa^	0.413	—	—	—
Postsurgery	79.91 ± 13.65^Aa^	78.20 ± 13.31^Aa^	0.597	—	—	—
*p*‐Value ^∗∗^	***0.043***	0.128	—	—	—	—
Respiratory rate						
Presurgical	17.41 ± 2.15^Aa^	17.40 ± 1.47^Aa^	0.719	0.156	0.337	0.348
Trans‐surgical	18.18 ± 1.94^Aa^	18.20 ± 2.48^Aa^	0.768	—	—	—
Postsurgery	18.00 ± 1.41^Aa^	16.95 ± 1.15^Ba^	***0.015***	—	—	—
*p*‐Value ^∗∗^	0.206	0.113	—	—	—	—
Temperature						
Presurgical	36.25 ± 0.60^Aa^	36.34 ± 0.50^Aa^	0.752	0.827	0.157	0.764
Trans‐surgical	36.22 ± 0.66^Aa^	36.25 ± 0.63^Aa^	0.890	—	—	—
Postsurgery	36.13 ± 0.58^Aa^	36.16 ± 0.54^Aa^	0.970	—	—	—
*p*‐Value ^∗∗^	0.937	0.570	—	—	—	—
O_2_ saturation						
Presurgical	97.00 ± 3.59^Aa^	97.90 ± 0.91^Aa^	0.770	0.172	0.397	0.226
Trans‐surgical	97.36 ± 2.54^Aa^	97.00 ± 6.64^Aa^	0.174	—	—	—
Postsurgery	97.50 ± 2.74^Aa^	98.00 ± 1.08^Aa^	0.811	—	—	—
*p*‐Value ^∗∗^	0.762	0.191	—	—	—	—
VAS						
T1 (immediate postoperative)	6.82 ± 2.04^Aa^	6.05 ± 1.73^Ba^	***0.035***	0.414	***<0.001***	0.572
T2 (postoperative 12 h)	5.82 ± 2.72^Ab^	4.95 ± 2.14^Ab^	0.155	—	—	—
T3 (postoperative 24 h)	4.64 ± 2.34^Ab^	3.80 ± 1.96^Ab^	0.259	—	—	—
*p*‐Value ^∗∗^	***<0.001***	***<0.001***	—	—	—	—

*Note:* Different uppercase letters = *p*  < 0.05 between groups; different lowercase letters = significant difference between periods. For the assessment of the time × group interaction, a two‐way repeated‐measures ANOVA was used for parametric data, while a generalized linear model (GLM) was used for nonparametric data. Values in bold and italics indicate statistically significant differences.

^∗^Mann–Whitney test.

^∗∗^Friedman/Dunn test (mean ± SD).

The heart rate showed no significant variation between the groups or difference between them. However, despite not varying significantly throughout the periods in the two study groups, the respiratory rate was significantly higher in the control group than in the music therapy group (*p* = 0.015). Temperature and O2 saturation did not differ significantly between the groups or over the evaluated periods (Table 3).

Regarding pain perception, both in the control group (*p* < 0.001) and in the music therapy group (*p*  < 0.001), there was a significant increase in the 12‐h postoperative period (T2) and a decrease in the 24‐h postoperative period (T3), but in the immediate postoperative period (T1), the music therapy group presented lower pain scores than the control group (*p* = 0.035).

The mean time to the first rescue medication was significantly shorter in the group without music therapy compared with the music therapy group (3.31 ± 0.13 vs. 104.00 ± 24.63 h, respectively) (*p* < 0.001) (Figure 2).

**Figure 2:**
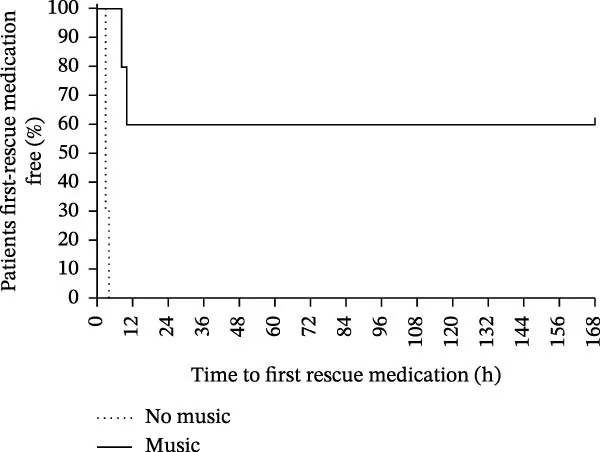
Comparison of rescue medication use and time to first rescue medication.

The mean number of tablets taken in the music therapy group was significantly lower than that in the group without music therapy (0.60 ± 0.84 vs. 6.32 ± 1.09, respectively) (*p* < 0.001).

The DAS scale showed no significant variation or difference between the groups (Table 4), as did the OHIP‐14 scale (Table 5).

**Table 4:** Dental anxiety scale (DAS).

DAS (items)	Pre		*p*‐Value^a^	Post		*p*‐Value^a^	*p*‐Value^b^	*p*‐Value^c^
	Control	Music therapy		Control	Music therapy			
How do you feel about coming to the dentist today?								
I think I am uncomfortable and will feel pain	4 (19.0%)	1 (4.8%)	0.101	3 (14.3%)	3 (14.3%)	0.837	0.976	0.229
I am terrified of what the dentist will say	1 (4.8%)	0 (0.0%)	—	1 (4.8%)	0 (0.0%)	—	—	—
I am feeling slightly uncomfortable	2 (9.5%)	8 (38.1%)	—	2 (9.5%)	3 (14.3%)	—	—	—
I do not mind	0 (0.0%)	1 (4.8%)	—	2 (9.5%)	3 (14.3%)	—	—	—
I am expecting a reasonably pleasant experience	14 (66.7%)	11 (52.4%)	—	13 (61.9%)	12 (57.1%)	—	—	—
How do you feel when you are waiting in the dentist’s waiting room?								
Tension	5 (23.8%)	5 (23.8%)	0.108	6 (28.6%)	3 (14.3%)	0.196	0.429	0.539
Looking forward to	7 (33.3%)	4 (19.0%)	—	4 (19.0%)	4 (19.0%)	—	—	—
Quite uncomfortable	0 (0.0%)	5 (23.8%)	—	3 (14.3%)	9 (42.9%)	—	—	—
Relaxed	9 (42.9%)	7 (33.3%)	—	8 (38.1%)	5 (23.8%)	—	—	—
How do you feel when you are in the dentist’s chair waiting for the dentist to prepare the motor to work on your teeth?								
So anxious that I start to sweat or feel bad	0 (0.0%)	1 (4.8%)	0.825	2 (9.5%)	0 (0.0%)	0.629	0.378	0.651
Tension	9 (42.9%)	9 (42.9%)	—	7 (33.3%)	6 (28.6%)	—	—	—
Looking forward to	4 (19.0%)	5 (23.8%)	—	4 (19.0%)	4 (19.0%)	—	—	—
Quite uncomfortable	2 (9.5%)	2 (9.5%)	—	4 (19.0%)	5 (23.8%)	—	—	—
Relaxed	6 (28.6%)	4 (19.0%)	—	4 (19.0%)	6 (28.6%)	—	—	—
You are in the dental chair when the dentist takes an instrument and uses it near your gums. How do you feel?								
So anxious that I start to sweat or feel bad	0 (0.0%)	1 (4.8%)	0.124	2 (9.5%)	2 (9.5%)	0.855	0.260	0.960
Tension	11 (52.4%)	5 (23.8%)	—	5 (23.8%)	6 (28.6%)	—	—	—
Looking forward to	1 (4.8%)	5 (23.8%)	—	2 (9.5%)	4 (19.0%)	—	—	—
Quite uncomfortable	4 (19.0%)	7 (33.3%)	—	7 (33.3%)	6 (28.6%)	—	—	—
Relaxed	5 (23.8%)	3 (14.3%)	—	5 (23.8%)	3 (14.3%)	—	—	—
How would you feel about receiving an anesthetic injection in your gums, in a back tooth?								
So anxious that I start to sweat or feel bad	2 (9.5%)	2 (9.5%)	0.661	2 (9.5%)	3 (14.3%)	0.781	0.705	0.671
Tension	7 (33.3%)	6 (28.6%)	—	7 (33.3%)	6 (28.6%)	—	—	—
Looking forward to	2 (9.5%)	2 (9.5%)	—	0 (0.0%)	1 (4.8%)	—	—	—
Quite uncomfortable	4 (19.0%)	8 (38.1%)	—	6 (28.6%)	4 (19.0%)	—	—	—
Relaxed	6 (28.6%)	3 (14.3%)	—	6 (28.6%)	7 (33.3%)	—	—	—

*Note: p*<0.05, Fisher’s exact test or Pearson’s chi‐square test.

^a^Control vs. music therapy.

^b^Control (pre vs. post).

^c^Music therapy (pre vs. post) (*n*, %).

**Table 5:** Oral health impact profile.

OHIP‐14 (items)	Group		*p*‐Value	Δ (CI95%)
	Control	Music therapy		
1. Have you had difficulty pronouncing any words because of problems with your teeth, mouth, or dentures?	0.50 ± 0.80	0.30 ± 0.66	0.377	−0.24 (−0.22 to 0.70)
2. Have you been experiencing altered taste due to problems with your teeth, mouth, or dentures?	0.45 ± 0.91	0.30 ± 0.66	0.725	−0.19 (−0.31 to 0.69)
3. Has there been severe pain in your mouth?	1.68 ± 0.99	1.80 ± 1.15	0.675	+0.14 (−0.81 to 0.53)
4. Have you been bothered by eating any food?	2.09 ± 1.23	1.65 ± 1.09	0.256	−0.33 (−0.40 to 1.07)
5. Have you felt embarrassed by problems with your teeth, mouth, or dentures?	1.91 ± 1.31	1.90 ± 1.45	0.938	−0.00 (−0.86 to 0.86)
6. Have you felt tense because of a problem with your teeth, mouth, or dentures?	1.55 ± 1.41	1.50 ± 1.24	0.990	−0.19 (−0.64 to 1.02)
7. Has your diet been unsatisfactory due to problems with your teeth, mouth, or dentures?	1.00 ± 1.23	0.95 ± 1.00	0.957	−0.14 (−0.56 to 0.85)
8. Have you had to stop eating because of problems with your teeth, mouth, or dentures?	0.41 ± 0.85	0.35 ± 0.67	0.960	−0.10 (−0.39 to 0.58)
9. Have you found it difficult to relax because of problems with your teeth, mouth, or dentures?	1.45 ± 1.06	1.20 ± 1.06	0.482	−0.19 (−0.47 to 0.85)
10. Have you felt a little embarrassed by problems with your teeth, mouth, or dentures?	0.77 ± 0.92	0.70 ± 1.13	0.526	−0.14 (−0.50 to 0.78)
11. Have you felt a little irritated with other people because of problems with your teeth, mouth, or dentures?	1.05 ± 1.09	0.80 ± 1.01	0.460	−0.33 (−0.32 to 0.98)
12. Have you had difficulty performing daily tasks because of problems with your teeth, mouth, or dentures?	0.64 ± 0.85	0.85 ± 0.88	0.388	+0.14 (−0.68 to 0.40)
13. Have you felt less satisfaction with life in general because of problems with your teeth, mouth, or dentures?	0.68 ± 0.84	0.65 ± 0.93	0.789	−0.10 (−0.46 to 0.65)
14. Have you felt totally incapable because of problems with your teeth, mouth, or dentures?	0.18 ± 0.39	0.30 ± 0.57	0.548	+0.10 (−0.40 to 0.21)
OHIP‐14 (domains)				
Functional limitation	0.95 ± 1.40	0.60 ± 1.23	0.258	−0.43 (−0.39 to 1.25)
Physical pain	3.77 ± 1.88	3.45 ± 1.93	0.683	−0.19 (−1.00 to 1.38)
Psychological discomfort	3.45 ± 2.26	3.40 ± 2.23	0.970	−0.19 (−1.21 to 1.59)
Physical disability	1.41 ± 1.97	1.30 ± 1.34	0.739	−0.24 (−0.82 to 1.30)
Psychological disability	2.23 ± 1.34	1.90 ± 1.80	0.262	−0.33 (−0.65 to 1.32)
Social disability	1.68 ± 1.78	1.65 ± 1.66	0.968	−0.19 (−0.89 to 1.27)
Handicap	0.86 ± 1.08	0.95 ± 1.19	0.848	−0.00 (−0.71 to 0.71)
OHIP‐14 (overall score)	14.36 ± 8.97	13.25 ± 8.15	0.705	−1.57 (−3.77 to 6.92)

*Note:* *p* < 0.05, Mann–Whitney test (mean ± SD).

## 4. Discussion

The study’s results suggested that music therapy contributed to a decrease in systolic pressure, a decrease in pain, and a decrease in the respiratory rate in the patients in the group that underwent the intervention. These conclusions were based on the changes in vital signs observed in patients who were submitted and not submitted to music therapy during the mandibular third molar exodontia surgical procedure, thus showing a positive impact from this use.

Corroborating the present study, Brazoloto [16] reported that music is capable of interfering positively with vital signs and in the perception of pain in patients, with a reduction in pain and a decrease in blood pressure and respiratory rate since we obtained results regarding the perception of pain in the T1 music therapy group that presented lower pain scores than the control group, with lower systolic blood pressure values and a more controlled respiratory rate in the operative times.

In a randomized clinical trial study conducted by Kupeli and Gülnahar [2], which included 80 patients, 44 females and 36 males, aged between 18 and 30 years, evaluated different music genres to reduce dental anxiety, but found no significant difference between groups regarding anxiety levels and heart rate. There were also no significant differences in the clinical characteristics between the two study groups, thus diverging from our study since the mean systolic pressure of the control patients was significantly higher than that of the patients under music therapy. The respiratory rate was significantly higher in the control group compared to the music therapy group. However, the present study agrees that there were no significant differences in clinical characteristics between the two study groups.

Consolidating the present study, Aravena et al. [4] reported that music therapy is a nonpharmacological method that can have a suppressive action on the sympathetic nervous system, which makes the patient have an adrenergic reduction in the body, thus reducing anxiety levels. Through a randomized clinical trial with the participation of 42 patients, it was concluded that drug music therapy could be considered a noninvasive, economical, and affective intervention to produce lower anxiety levels in the patient.

According to Packyanathan et al. [3], in a study of 50 patients who visited the Saveetha Dental College outpatient clinic for dental extractions, some were randomly selected and allocated to the test group and control group to evaluate the effects of music therapy on dental anxiety and hemodynamic changes, namely, systolic pressure, diastolic pressure, and heart rate. This evaluation was performed before and after the extraction. The study showed that the control population had elevated hemodynamic changes concerning systolic and diastolic blood pressure and heart rate, with the elevation of diastolic pressure being significant. In the test group, there was a drop in hemodynamic changes concerning systolic and diastolic blood pressure and heart rate, all of which were statistically significant. This was also evident on the DAS. This study conflicts with our study since, in our research, no major significant variations in systolic or diastolic blood pressure were evident, and there was no significant variation or difference between the groups in the DAS as well as in the OHIP‐14 scale.

Liu and Petrini [17] conducted a study with 112 patients in the thoracic surgery department of tertiary hospitals in Wuhan, China, using music therapy in the preoperative and postoperative periods and were able to find that there was a statistically significant decrease in systolic blood pressure, heart rate, pain, and anxiety in the research participants. This study concluded that there was no significant difference in diastolic blood pressure and respiratory rate.

The present research also agrees with the findings in the literature described by Liu and Petrini [17] from the statistical results of the sample of this study that used music therapy during dental surgical procedures, it was concluded that there were reductions in respiratory rate and systolic blood pressure, and disagrees about the decrease in diastolic blood pressure and a significant reduction in respiratory rate when comparing the groups submitted and not submitted to music therapy. This study is also contradictory because the patients were submitted to the efficacy of a 3‐day music intervention on pain, anxiety, and vital signs after thoracic surgery, while our research evaluated this efficacy in the transoperative and postoperative periods.

It is known that the physiological system is responsible for adjusting blood pressure by changing heart rate, blood volume, and peripheral resistance. According to Yamashita et al. [7], music, when listened to, activates areas of the brain capable of generating feelings of pleasure and motivation, which would explain the positive results of our research when we relate the decrease in systolic pressure with the decrease in heart rate.

Monteiro et al. [18] reported in the systematic review that music may be an effective nonpharmacological measure to reduce preoperative anxiety in patients scheduled for third molar extraction. Therefore, music may be introduced from the perspective of humanistic medicine, corroborating the findings of this clinical trial.

The limitations of our study lie in the need to conduct it on a larger sample size and to include additional physiological parameters. Anxiety fluctuates over time and is influenced by environment, interpersonal relationships, and past experience. Effectively isolating and measuring the effect of a single variable, such as music on anxiety, requires exquisitely controlled settings. Furthermore, other key limitations include the impossibility of blinding participants to the auditory intervention, the reliance on subjective, self‐reported pain assessments, and the single‐center design, which may limit the generalizability of our findings to broader populations or different clinical settings.

This study provides more evidence to support the practice of music therapy with the aim of reducing pain and anxiety and, consequently, increasing the quality of life of patients when undergoing lower third molar extraction surgery.

Pain and anxiety are directly correlated and can induce the production of endogenous catecholamines, increasing the negative effects on the cardiovascular and respiratory systems in patients undergoing dental surgery. In this scenario, music therapy becomes a nonpharmacological, very economical, and effective method of making the patient more relaxed, being able to promote a decrease in systolic pressure, alleviating preoperative anxiety, and even decreasing the patients respiratory rate, being able to avoid unwanted complications related to these parameters.

It is important to emphasize the importance of the patient choosing the musical genre they are interested in listening to, always remembering that the musical tastes of one individual may be different from another, and that familiarization with their favorite songs can contribute to relaxation, reducing fear, pain, and anxiety during the surgical procedure.

## 5. Conclusion

Music therapy during lower third molar extraction was associated with lower systolic blood pressure and respiratory rate as well as lower pain scores in the immediate postoperative period. No significant differences were observed between the groups regarding anxiety (DAS) or oral health‐related quality of life (OHIP‐14). These findings suggest that music therapy may have beneficial effects on selected physiological parameters, specifically systolic blood pressure and respiratory rate, as well as on pain perception in the immediate postoperative period, although its effects on anxiety and oral health‐related quality of life were not demonstrated in the present study.

## Funding

This research did not receive any specific grants or aid from funding agencies in the public, commercial, or nonprofit sectors.

## Ethics Statement

Approval was given by the Ethics Committee, under protocol number 4,928,668.

## Consent

All patients signed the consent form and agreed to participate in this study.

## Conflicts of Interest

The authors declare no conflicts of interest.

## Data Availability

The data that support the findings of this study are available from the corresponding author upon reasonable request.
